# Atypical presentation of an unusual foreign body

**DOI:** 10.4103/0301-4738.64138

**Published:** 2010

**Authors:** Vipul Arora, Usha R Kim, Shashikant Shetty, Akash D Shah

**Affiliations:** Aravind Eye Hospital, Madurai, Tamil Nadu, India

**Keywords:** Diplopia, intraorbital foreign body, pen injury

## Abstract

A 14-year-old boy presented with intractable diplopia for 10 days following an assault. A thorough history revealed that he was unaware of any penetrating injury. However, imaging demonstrated a radiolucent foreign body between the globe and the orbital floor. On surgical exploration, it was found to be the proximal part of a ball point pen. Its removal resulted in complete resolution of diplopia. Thorough clinical and radiological examination is recommended when a foreign body is suspected in pediatric patients. Prompt diagnosis will aid in early intervention and prevention of long-term complications.

The most common orbital foreign bodies following penetrating orbital injury are glass and wood.[1‐4] Most are detected because of clinical suspicion due to an obvious external entrance wound. We report a case of penetrating injury in the orbit with no obvious wound. This injury went unnoticed by the patient at the time of the trauma, but later presented with diplopia and hyperglobus.

## Case Report

**Figure 1:**
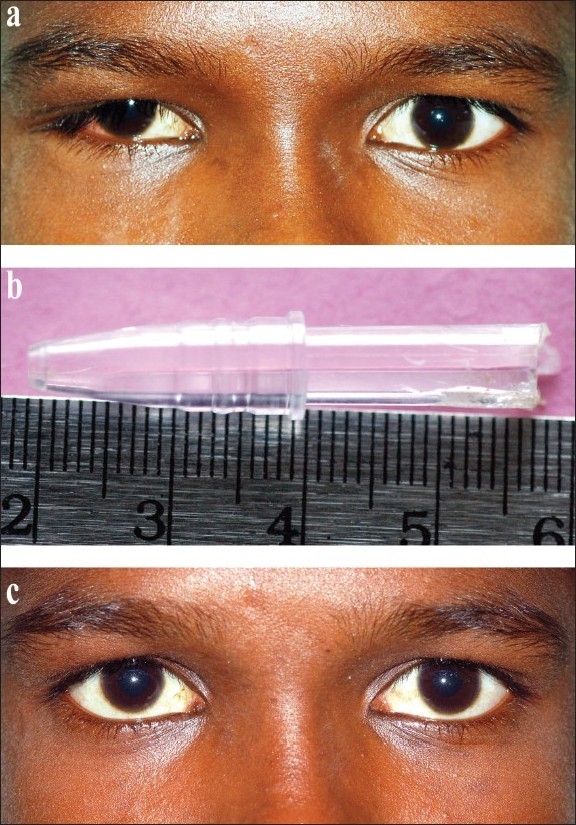
(a) Clinical photograph of patient shows less than 15 degrees of hypotropia in the right eye on Hirschberg test (b) Plastic pen tip measuring 39 × 4 mm removed from the floor of orbit. (c) Postoperative clinical photograph of patient showing small residual hypotropia in the right eye

A 14-year-old boy presented with double vision following an assault by a friend 10 days before presenting to our clinic. Further questioning revealed that his friend had hit him in his right eye with a pen. The patient denied any significant trauma to the eye. When examined, the patient had a left face turn. The visual acuity was 20/20 in both eyes. There were no signs of injury in the external right eyelid [Fig 1a]. However, there was a conjunctival tear inferolaterally. There was a firm mass palpable along the inferotemporal orbital fossa between the globe and the orbital rim. There was less than 15 degrees of hypotropia on Hirschberg's test, while prism bar cover test revealed hypotropia of 20-prism diopter in the right eye [Fig 1a]. Extraocular movements were restricted in upgaze as well as downgaze. Diplopia charting showed vertical diplopia in primary gaze, upgaze and downgaze. Hess chart findings were suggestive of maximum underaction in the area of the right inferior oblique as well as underaction in the area of the right inferior rectus, with overaction in the left eye. Force duction test in the right eye revealed restriction of elevation and depression. Anterior segment and fundus examination was normal with a brisk pupillary reaction.

**Figure 2:**
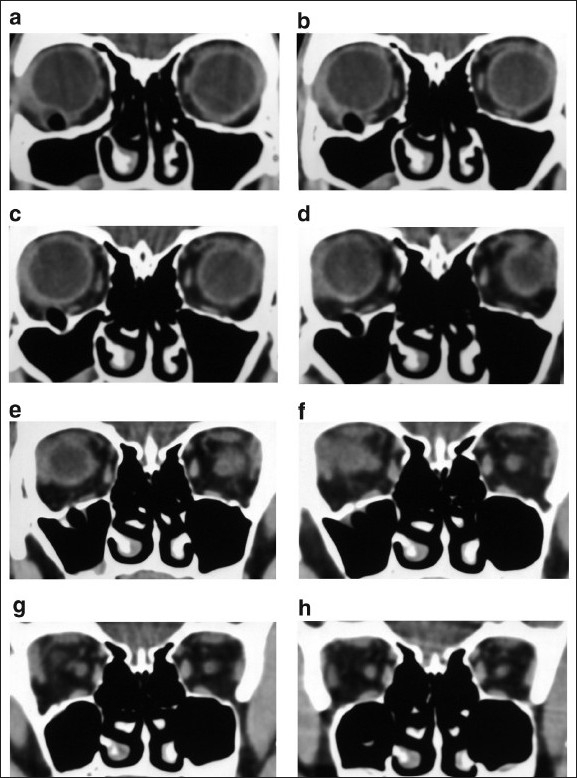
(a-h) Sequential coronal computed tomography scan of patient showing well-defined radiolucent foreign body passing posteriorly and caudally in maxillary sinus with orbital floor fracture

A computed tomography (CT) scan showed a well-defined tubular translucent object which was indenting the sclera in the right eye inferolaterally and passed inferomedially and posteriorly. There was a defect of the floor of the right orbit with the foreign body extending inferiorly into the maxillary antrum across the fracture site [Fig 2 a-h].

Exploration of the right orbit via trans-conjunctival approach was planned. After making an incision in the conjunctiva, a solid structure was appreciated. This was a plastic transparent tip of a ball point pen [Fig 1b]. It was removed without damaging the inferior oblique or inferior rectus muscle fibers. The tip of the pen entered the maxillary sinus posteriorly, where a circumscribed defect was noticed in the orbital floor. As there was no entrapment at the fracture site, no plating was done. The patient was given systemic oral steroids (prednisolone acetate; 1 mg/kg body weight in tapering dose) postoperatively for one month.

On one month follow-up there was small residual hypotropia. However, he was able to fuse and therefore had no complaint of diplopia in primary gaze [Fig 1c].

## Discussion

Intraorbital foreign body usually presents with a history of high-velocity injuries, for example industrial accidents or gunshot injuries. However, in some cases of trivial trauma patients are not aware of any penetrating injury.[2] These occult foreign bodies that penetrate the orbit are only detected when a secondary complication arises.[5] These complications vary from visual loss, severe orbital inflammation, secondary infection, osteomyelitis, ptosis and even brain abscess.[5,6]

A complaint of diplopia in orbital trauma is commonly seen in cases of an orbital floor fracture, which is mainly due to inferior rectus entrapment or rarely from nerve injury or muscle contusion. However, this presentation is rare in penetrating injury by an intraorbital foreign body because of visual compromise caused by associated trauma to the globe.[7,8] Our case was unique as the patient was unaware of any external object penetrating the eye; and the alignment of the proximal portion of the pen between the globe and orbital floor was unusual. This caused restriction in the movement of the globe, leading to diplopia. Removal of the foreign body was followed by resolution of double vision with no restriction in extraocular movement. To the best of our knowledge, restrictive strabismus causing diplopia due to retained intraorbital foreign body has not been reported in literature. Our case also highlights the significance of conjunctival tear, which, when it extends inferolaterally with no posterior extent, warrants careful clinical examination and exploration.

Imaging plays a vital role in the evaluation of the globe and orbital integrity following trauma. Although X-ray is the initial imaging that can be done to confirm a foreign body, CT scan is the investigation of choice when there is a definitive history of penetrating injury or when a clinician suspects any intraorbital foreign body. In cases of a injury with a wooden foreign body, magnetic resonance imaging (MRI) is preferred as the latter may be missed on CT imaging, mimicking intraorbital air.[2] This false perception may also occur in cases presenting with injury with a plastic object.[9] These radiolucent foreign bodies may be missed, if imaging is not correlated with the clinical examination.

Management of the intraorbital foreign body depends on clinical presentation, nature and the location of the foreign body in the orbit. Presentation of the patient with visual compromise, ptosis, diplopia, orbital inflammation or infection demands its removal. All organic foreign bodies should be removed as they may later cause complications. However, in cases of inorganic foreign bodies, surgical exploration depends on their location. Anteriorly located foreign bodies can easily be removed, whereas foreign bodies located more posteriorly without any clinical features should be left as such, as their removal may result in grave complications.[2]

Clinical history may sometimes be deceptive, especially in children, therefore care must be taken to correlate it with thorough clinical examination, appropriate imaging and surgical exploration when required.
